# Supplementary material for: Unveiling the Bioactive Potential of the Invasive Jellyfish Phyllorhiza punctata Through Integrative Transcriptomic and Proteomic Analyses
Source: Biomolecules. 2025 Aug 4;15(8):1121. doi: 10.3390/biom15081121 (PMC12383608; doi:10.3390/biom15081121)
Supplement: Supplementary file 1 [file biomolecules-15-01121-s001.zip › Suplementary Material/FileS3/PEPFOLD4/XP_065068888_8906/clusters.html]

 PEP-FOLD clusters


Download archive of all models  here   

|  |  |  |
| --- | --- | --- |
| # | file | sOPEP |
| # cluster 1 - Size: 3 |  |  |
|  | XP\_065068888\_8906-model1 | -15.7305 |
|  | XP\_065068888\_8906-model1.1 | -11.1549 |
|  | XP\_065068888\_8906-model1.2 | -9.79062 |
| # cluster 2 - Size: 1 |  |  |
|  | XP\_065068888\_8906-model2 | -11.9881 |
| # cluster 3 - Size: 11 |  |  |
|  | XP\_065068888\_8906-model3 | -11.7628 |
|  | XP\_065068888\_8906-model3.1 | -11.5728 |
|  | XP\_065068888\_8906-model3.2 | -10.9306 |
|  | XP\_065068888\_8906-model3.3 | -10.9045 |
|  | XP\_065068888\_8906-model3.4 | -10.7859 |
|  | XP\_065068888\_8906-model3.5 | -10.7078 |
|  | XP\_065068888\_8906-model3.6 | -10.7037 |
|  | XP\_065068888\_8906-model3.7 | -10.5758 |
|  | XP\_065068888\_8906-model3.8 | -10.5019 |
|  | XP\_065068888\_8906-model3.9 | -10.099 |
|  | XP\_065068888\_8906-model3.10 | -9.3743 |
| # cluster 4 - Size: 5 |  |  |
|  | XP\_065068888\_8906-model4 | -11.6084 |
|  | XP\_065068888\_8906-model4.1 | -11.5891 |
|  | XP\_065068888\_8906-model4.2 | -10.9941 |
|  | XP\_065068888\_8906-model4.3 | -10.5857 |
|  | XP\_065068888\_8906-model4.4 | -10.0647 |
| # cluster 5 - Size: 4 |  |  |
|  | XP\_065068888\_8906-model5 | -11.5865 |
|  | XP\_065068888\_8906-model5.1 | -10.3707 |
|  | XP\_065068888\_8906-model5.2 | -10.1398 |
|  | XP\_065068888\_8906-model5.3 | -9.90866 |
| # cluster 6 - Size: 6 |  |  |
|  | XP\_065068888\_8906-model6 | -11.52 |
|  | XP\_065068888\_8906-model6.1 | -11.2341 |
|  | XP\_065068888\_8906-model6.2 | -11.216 |
|  | XP\_065068888\_8906-model6.3 | -11.0368 |
|  | XP\_065068888\_8906-model6.4 | -10.5312 |
|  | XP\_065068888\_8906-model6.5 | -10.4884 |
| # cluster 7 - Size: 3 |  |  |
|  | XP\_065068888\_8906-model7 | -11.3846 |
|  | XP\_065068888\_8906-model7.1 | -11.2601 |
|  | XP\_065068888\_8906-model7.2 | -10.4602 |
| # cluster 8 - Size: 15 |  |  |
|  | XP\_065068888\_8906-model8 | -11.3108 |
|  | XP\_065068888\_8906-model8.1 | -11.1157 |
|  | XP\_065068888\_8906-model8.2 | -10.9333 |
|  | XP\_065068888\_8906-model8.3 | -10.8483 |
|  | XP\_065068888\_8906-model8.4 | -10.6695 |
|  | XP\_065068888\_8906-model8.5 | -10.6192 |
|  | XP\_065068888\_8906-model8.6 | -10.5967 |
|  | XP\_065068888\_8906-model8.7 | -10.5384 |
|  | XP\_065068888\_8906-model8.8 | -10.537 |
|  | XP\_065068888\_8906-model8.9 | -10.4605 |
|  | XP\_065068888\_8906-model8.10 | -10.3861 |
|  | XP\_065068888\_8906-model8.11 | -10.3126 |
|  | XP\_065068888\_8906-model8.12 | -10.2024 |
|  | XP\_065068888\_8906-model8.13 | -10.1365 |
|  | XP\_065068888\_8906-model8.14 | -9.46173 |
| # cluster 9 - Size: 4 |  |  |
|  | XP\_065068888\_8906-model9 | -11.2749 |
|  | XP\_065068888\_8906-model9.1 | -11.0887 |
|  | XP\_065068888\_8906-model9.2 | -10.7405 |
|  | XP\_065068888\_8906-model9.3 | -10.7104 |
| # cluster 10 - Size: 4 |  |  |
|  | XP\_065068888\_8906-model10 | -11.1923 |
|  | XP\_065068888\_8906-model10.1 | -11.0896 |
|  | XP\_065068888\_8906-model10.2 | -9.64762 |
|  | XP\_065068888\_8906-model10.3 | -9.3968 |
| # cluster 11 - Size: 5 |  |  |
|  | XP\_065068888\_8906-model11 | -11.1737 |
|  | XP\_065068888\_8906-model11.1 | -11.1107 |
|  | XP\_065068888\_8906-model11.2 | -10.6091 |
|  | XP\_065068888\_8906-model11.3 | -10.4851 |
|  | XP\_065068888\_8906-model11.4 | -10.1763 |
| # cluster 12 - Size: 1 |  |  |
|  | XP\_065068888\_8906-model12 | -11.0636 |
| # cluster 13 - Size: 1 |  |  |
|  | XP\_065068888\_8906-model13 | -11.0148 |
| # cluster 14 - Size: 1 |  |  |
|  | XP\_065068888\_8906-model14 | -10.9061 |
| # cluster 15 - Size: 2 |  |  |
|  | XP\_065068888\_8906-model15 | -10.8968 |
|  | XP\_065068888\_8906-model15.1 | -9.97751 |
| # cluster 16 - Size: 5 |  |  |
|  | XP\_065068888\_8906-model16 | -10.8626 |
|  | XP\_065068888\_8906-model16.1 | -10.6768 |
|  | XP\_065068888\_8906-model16.2 | -10.239 |
|  | XP\_065068888\_8906-model16.3 | -9.87619 |
|  | XP\_065068888\_8906-model16.4 | -9.52101 |
| # cluster 17 - Size: 2 |  |  |
|  | XP\_065068888\_8906-model17 | -10.8535 |
|  | XP\_065068888\_8906-model17.1 | -10.3516 |
| # cluster 18 - Size: 1 |  |  |
|  | XP\_065068888\_8906-model18 | -10.7988 |
| # cluster 19 - Size: 1 |  |  |
|  | XP\_065068888\_8906-model19 | -10.7789 |
| # cluster 20 - Size: 3 |  |  |
|  | XP\_065068888\_8906-model20 | -10.5976 |
|  | XP\_065068888\_8906-model20.1 | -10.4767 |
|  | XP\_065068888\_8906-model20.2 | -10.2722 |
| # cluster 21 - Size: 1 |  |  |
|  | XP\_065068888\_8906-model21 | -10.5793 |
| # cluster 22 - Size: 1 |  |  |
|  | XP\_065068888\_8906-model22 | -10.5449 |
| # cluster 23 - Size: 2 |  |  |
|  | XP\_065068888\_8906-model23 | -10.5317 |
|  | XP\_065068888\_8906-model23.1 | -10.0371 |
| # cluster 24 - Size: 4 |  |  |
|  | XP\_065068888\_8906-model24 | -10.5223 |
|  | XP\_065068888\_8906-model24.1 | -10.3322 |
|  | XP\_065068888\_8906-model24.2 | -10.2239 |
|  | XP\_065068888\_8906-model24.3 | -9.97421 |
| # cluster 25 - Size: 2 |  |  |
|  | XP\_065068888\_8906-model25 | -10.5078 |
|  | XP\_065068888\_8906-model25.1 | -10.0936 |
| # cluster 26 - Size: 2 |  |  |
|  | XP\_065068888\_8906-model26 | -10.4378 |
|  | XP\_065068888\_8906-model26.1 | -9.7575 |
| # cluster 27 - Size: 1 |  |  |
|  | XP\_065068888\_8906-model27 | -10.3195 |
| # cluster 28 - Size: 1 |  |  |
|  | XP\_065068888\_8906-model28 | -10.0661 |
| # cluster 29 - Size: 1 |  |  |
|  | XP\_065068888\_8906-model29 | -10.0228 |
| # cluster 30 - Size: 1 |  |  |
|  | XP\_065068888\_8906-model30 | -10.0054 |
| # cluster 31 - Size: 1 |  |  |
|  | XP\_065068888\_8906-model31 | -9.82704 |
| # cluster 32 - Size: 2 |  |  |
|  | XP\_065068888\_8906-model32 | -9.68335 |
|  | XP\_065068888\_8906-model32.1 | -9.3514 |
| # cluster 33 - Size: 1 |  |  |
|  | XP\_065068888\_8906-model33 | -9.59796 |
| # cluster 34 - Size: 1 |  |  |
|  | XP\_065068888\_8906-model34 | -9.11711 |
| # cluster 35 - Size: 1 |  |  |
|  | XP\_065068888\_8906-model35 | -9.01769 |
|  |  |  |
